# Prolongation of the QT Interval and Post-Extrasystolic Augmentation of the TU-Wave During Emotional Stress

**Published:** 2008-04-01

**Authors:** Velislav N Batchvarov, Abhay Bajpai, Elijah Behr

**Affiliations:** Division of Cardiac and Vascular Sciences, St. George's University of London, London, United Kingdom

**Keywords:** long QT syndrome, QT prolongation, U wave, post-extrasystolic T wave changes

## Abstract

We present a case of a 25-year-old woman with multiple blackouts and no structural heart disease, with abnormal T-U waves and borderline QT interval on her resting electrocardiogram. During emotional stress she developed frequent monomorphic ventricular premature beats, with characteristic changes of the sinus complexes immediately following the premature beats, namely augmentation and greater degree of merging of the T and U waves and QTc interval prolongation. The changes alert about the possibility of congenital long QT syndrome, specifically genotype 2 or 1.

Figure 1 presents a resting electrocardiogram (ECG) recorded at 25 mm/sec, 10 mm/mV in a 25-year-old woman with a history of multiple blackouts. It shows sinus rhythm, heart rate of 80 beats per minute, notched T wave in lead V2, and abnormal augmented U wave in leads V2 to V4. The QT interval measured to the T-U nadir is 383 ms (manual measurement with on-screen calliper) and the QTc interval (correction by Bazett's formula) is borderline prolonged at 442 ms.

The ECG presented in Figure 2 was recorded in the same patient few minutes later, apparently during emotional stress (she was awaiting ajmaline test to exclude Brugada syndrome, and has just been told that there was a risk of 1 in 200 of inducing an arrhythmia during the test that would need an electrical shock to be stopped). The average heart rate is 106 beats/minute and every forth beat is a ventricular premature beat (VPB) (pattern of quadrigeminy). The T-U wave of each sinus beat immediately following a VPB is augmented and with a greater degree of T-U merging compared to the T-U wave of the next sinus beat. The QT interval of the 1st post-VPB sinus beat measured to the T-U wave nadir is 396 ms and the corrected QTc is prolonged at 528 ms, although precise measurement seems difficult.

In Figure 3 (right panel), the ST-TU waves of the 4 sinus complexes immediately following a VPB (black lines) and of the next 4 sinus beats (grey lines) have been superimposed to demonstrate the post-VPB augmentation of the T-U wave. In Figure 3, left panel, all sinus complexes of the resting ECG presented in Figure 1 have been superimposed for comparison.

The borderline QT prolongation with abnormal T and U waves in a symptomatic young female patient alerts about the possibility of congenital Long QT Syndrome [1]. The ECG during emotional stress displays features characteristic for this condition (specifically genotype 2 or 1 [2]): further prolongation of the QTc interval and augmentation of the abnormal U wave with T-U merging of the immediate post-extrasystolic sinus beats [3].

## Figures and Tables

**Figure 1:**
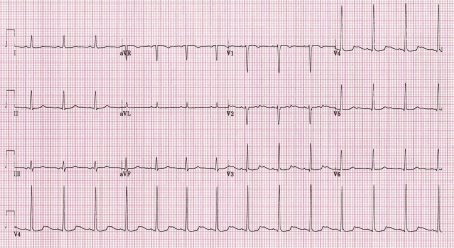
Resting ECG in a 25-year-old woman with daily blackouts. See the text for details

**Figure 2:**
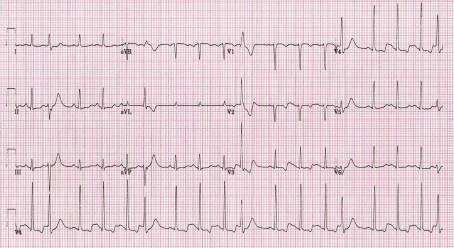
Resting ECG recorded in the same patient during emotional stress. See the text for details

**Figure 3:**
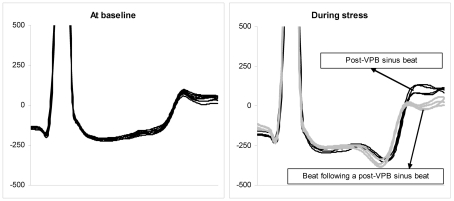
Left panel: All QRS-T complexes in lead V4 of the ECG presented in Figure 1 have been superimposed and aligned by the QRS complex. Right panel: The 4 QRS-T sinus complexes in lead V4 immediately following a ventricular premature beat (black lines) and the next 4 QRS-T sinus complexes (grey lines) have been superimposed and aligned by the QRS complex to demonstrate the difference in the T wave shape. The scale is arbitrary. See the text for details
